# Endometrial stromal sarcoma

**DOI:** 10.4103/0971-5851.68848

**Published:** 2010

**Authors:** Geetha Puliyath, V.Rajasekharan Nair, Swetha Singh

**Affiliations:** *Department of Obstetrics and Gynaecology, SUT Academy of Medical Sciences, Vattappara, Trivandrum, Kerala, India*

**Keywords:** *Endometrial stromal sarcoma*, *rapid enlargement of fibroid uterus*, *uterine sarcoma*

## Abstract

Endometrial stromal sarcoma (ESS) is a rare malignant tumor of the endometrium, occurring in the age group of 40–50 years. This is a case of low-grade ESS presenting as rapid enlargement of a fibroid uterus. Because of her secondary infertility, she was planned for myomectomy. However, due to the high degree of suspicion of a sarcomatous change in the fibroid, in view of rapid enlargement of uterus within the last 4 months, we planned for a preoperative endometrial aspiration. It showed low-grade ESS, which was later confirmed by histopathology examination of total hysterectomy specimen. As surgery was the main treatment for ESS, because of the proper preoperative diagnosis, we could plan the treatment accordingly. Despite the rarity of the tumor, one has to consider the possibility of ESS in cases with presentation of rapid enlargement of a fibroid uterus.

## INTRODUCTION

Cancers arising from mesodermal structures like muscles and connective tissue are called sarcomas. Sarcomas of the uterus are uncommon, and may arise from connective tissue, smooth muscle or the endometrial stroma. Uterine sarcoma is a rare form of malignancy, occurring in 2–5% of all patients with uterine malignancy, with an incidence of approximately one to two cases per 100,000 women in the general population. Endometrial stromal sarcomas (ESSs) are very rare malignant tumors that make up approximately 10% of all uterine sarcomas but only around 0.2% of all uterine malignancies.[1]

## CASE REPORT

**Figure 1:**
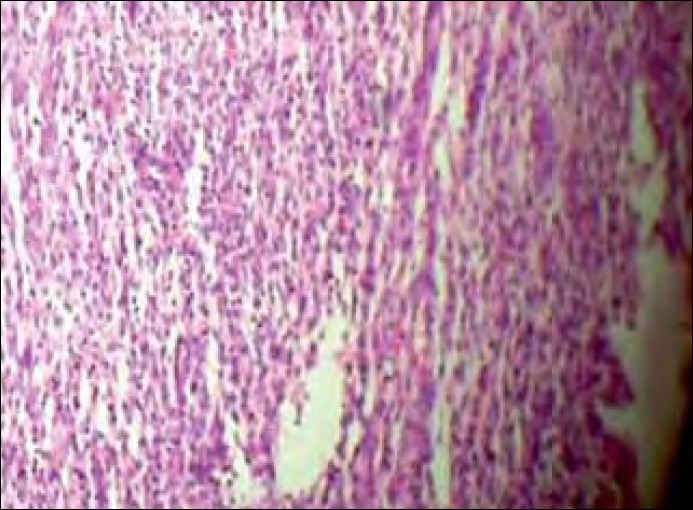
Endometrial aspiration: histopathological examination (HPE), endometrial glands in the secretory phase (arrow) with neoplastic cells suggestive of low-grade endometrial stromal sarcoma

A 30-year-old lady presented with increased bleeding per vagina during periods since 1 year. She was a Para1, Live1, with last child birth 10 years back and no history of contraceptive use. She had attained menarchy at the age of 14, and her previous cycles were normal. However, for the past 1 year, her cycles were prolonged, lasting for 15–20 days/2–3 months. She was investigated for secondary infertility in a local hospital 1 year back and was treated with clomiphene citrate for three to four cycles. A pelvic scan taken 11 months back showed normal-sized uterus with a fibroid 3.7 cm×3.4 cm in the anterior myometrium. Endometrial thickness was 8 mm. A dilatation and curettage was performed and microscopy showed a disordered proliferate endometrium. She was given symptomatic treatment for menorrhagia. An ultrasound scan carried out 4 months back, showed enlarged uterus (12 cm×7 cm×7 cm) with diffuse coarse heterogeneous myometrial echoes. A later scan showed further enlargement of the uterus (16 cm×13 cm×10 cm) with the intramural fibroid 10 cm×7 cm in posterior wall. She was advised myomectomy and was referred to our tertiary care institute. Her general and systemic examinations were normal. Abdominal and pelvic examinations revealed a uniformly enlarged uterus, 24 weeks size, firm in consistency, with restricted mobility. Doppler ultrasound showed a fibroid uterus 16 cm and 12 cm, with normal vascularity. Endometrial aspiration was performed due to suspected sarcomatous changes in view of the rapid enlargement of the uterus within the past 4 months. Microscopy showed endometrial glands in the secretory phase with neoplastic cells, suggestive of low-grade endometrial stromal sarcoma (LGESS) [Figure 1].

**Figure 2:**
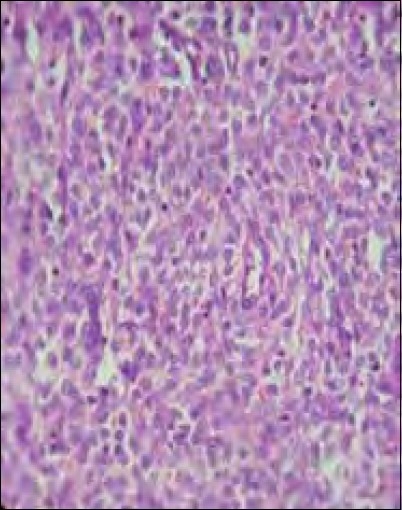
HPE (high power) showing uniform oval or spindle-shaped cells

**Figure 3:**
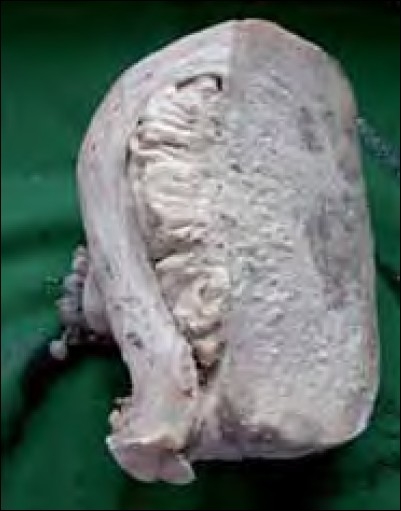
Tumor (arrow) almost perforating right side of the myometrium

**Figure 4:**
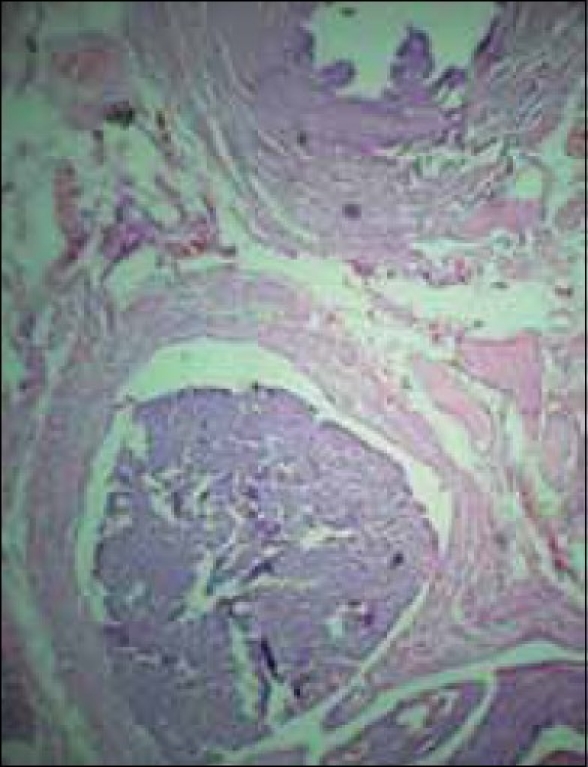
Section from the fallopian tube showing neoplastic cells in dilated lymphatic spaces

A total abdominal hysterectomy with bilateral salpingooopherectomy was performed through a vertical midline incision. The findings were a uniformly enlarged uterus with normal-looking tubes and ovaries The tumor had infiltrated the myometrium anteriorly. There were no metastatic deposits elsewhere. The lymph nodes were not enlarged. Grossly, the specimen had a yellowish tan. Microscopically, no malignant cells could be demonstrated in the peritoneal washings. Characteristically uniform oval and spindle-shaped cells, suggestive of low-grade ESS [Figure 2], infiltrating the entire thickness of the myometrium, were noted [Figure 3]. A section from the right fallopian tube showed neoplastic cells in dilated lymphatic spaces [Figure 4]. The cervix and ovaries were normal. The case was confirmed to be a low-grade endometrial stromal sarcoma stage 3, and she was referred to a regional cancer center.

## DISCUSSION

ESSs are very rare malignant tumors. Based on tumor margin status and cytological features, the WHO has classified endometrial stromal tumor into benign endometrial stromal nodule (ESN) and endometrial stromal sarcoma. ESN does not infiltrate the myometrium. They are well circumscribed, with a pushing margin. ESSs infiltrate the myometrium and are characterized by proliferation of uniform small cells closely resembling those of endometrial stroma in the proliferative stage.[2] ESS can be divided into low-grade and high-grade tumors according to cell morphology and mitotic count. Low-grade ESS usually occur in the young population (mean age 39 years), contrary to the high-grade ESS (mean age 61 years). Low-grade ESSs have less-frequent mitosis (<3 per 10 high-power fields) and they do not show hemorrhage and necrosis.[3] Some authors have regarded high grade endometrial stromal sarcoma (HGESS) as an undifferentiated sarcoma.[4]

Most ESSs involve the endometrium, and uterine curettage usually leads to diagnosis.[2] The main differential diagnosis of low-grade ESS includes ESN, cellular leiomyoma and cellular intravenous leiomyomatosis.[5] The microscopic appearance of ESS and ESN are identical. Infiltrative margins and distinctive growth as worm-like cords are noted in low-grade ESS, whereas the margins are well demarcated in ESN. Hence, extensive sampling of tumor margins and detecting vascular invasion are extremely important in distinguishing between the two. Cellular leiomyomas are composed of cells with spindle-shaped nuclei with a fascicular growth pattern, thick muscular-walled vessels, cleft-like spaces and showing focal merging with the adjacent myometrium.[6] In low-grade ESS, cords of tumor cells infiltrate between smooth muscle and within lymphatic spaces. The neoplastic stromal cells resemble those of the proliferative endometrium, are monotonous in appearance and have a uniform size and shape. The nuclei are round to ovoid, with fine chromatin, and a small nucleoli is seen. A small amount of cytoplasm is present and cell borders are indistinct. Mitotic activity is low (<10/10 high-power fields).[5] About a third to a half of the low-grade ESS have extrauterine spread at the time of diagnosis. When there is a difficulty in diagnosing between ESS and cellular leiomyoma, immunoreactivity with antibodies to CD10 and smooth muscle actin and desmin are used.[7]

Surgery is the final resort for diagnosis and primary treatment of ESS. Preoperative diagnosis is mostly a uterine fibroid. Ultrasound and magnetic resonance imaging are inconclusive. If the diagnosis is known, the extent of surgery is planned according to the stage of the tumor. The FIGO staging for carcinoma of the corpus uteri has been applied to ESS.

The treatment is total abdominal hysterectomy, bilateral salpingooophorectomy and pelvic and periaortic selective lymphadenectomy. Cytologic washings are obtained from the pelvis and abdomen. Hormone therapy with medroxy progesterone, tamoxifen, gonadotropin releasing hormone (GnRH) analogues and aromatase inhibitors are suggested for low-grade ESS stage 3–4 and for recurrent disease.[8,9]

## CONCLUSION

Because of the rarity of the tumor, ESS may not be familiar to the gynaecologists. In young patients, it could be mistaken for a fibroid and more conservative management may have been planned accordingly. High degree of suspicion in a rapidly enlarged uterus led to an endometrial aspiration in this case, which showed low-grade ESS. Therefore, the treatment plan was shifted from a conservative myomectomy to a laparotomy, total abdominal hysterectomy and bilateral salpingo-opherectomy. The case highlights the necessity for high degree of suspicion and proper preoperative diagnosis in this rare type of tumor, especially in young patients.
